# Fixel-Based Analysis of White Matter Degeneration in Patients With Progressive Supranuclear Palsy or Multiple System Atrophy, as Compared to Parkinson's Disease

**DOI:** 10.3389/fnagi.2021.625874

**Published:** 2021-03-16

**Authors:** Thanh-Thao Nguyen, Jur-Shan Cheng, Yao-Liang Chen, Yu-Chun Lin, Chih-Chien Tsai, Chin-Song Lu, Yi-Hsin Weng, Yi-Ming Wu, Ngoc-Thanh Hoang, Jiun-Jie Wang

**Affiliations:** ^1^Department of Radiology, Hue University of Medicine and Pharmacy, Hue University, Hue, Vietnam; ^2^Clinical Informatics and Medical Statistics Research Center, College of Medicine, Chang Gung University, Taoyuan, Taiwan; ^3^Department of Biomedical Sciences, College of Medicine, Chang Gung University, Taoyuan, Taiwan; ^4^Department of Emergency Medicine, Chang Gung Memorial Hospital, Keelung, Taiwan; ^5^Department of Diagnostic Radiology, Chang Gung Memorial Hospital, Keelung, Taiwan; ^6^Department of Medical Imaging and Intervention, Chang Gung Memorial Hospital, Linkou, Taiwan; ^7^Department of Medical Imaging and Radiological Sciences, Chang Gung University, Taoyuan, Taiwan; ^8^Healthy Aging Research Center, Chang Gung University, Taoyuan, Taiwan; ^9^Professor Lu Neurological Clinic, Taoyuan, Taiwan; ^10^Division of Movement Disorders, Department of Neurology, Chang Gung Memorial Hospital, Linkou, Taiwan; ^11^Neuroscience Research Center, Chang Gung Memorial Hospital, Linkou, Taiwan; ^12^School of Medicine, Chang Gung University, Taoyuan, Taiwan; ^13^Medical Imaging Research Center, Institute for Radiological Research, Chang Gung University/Chang Gung Memorial Hospital, Linkou, Taiwan

**Keywords:** fixel-based analysis, white matter, Parkinson's disease, multiple system atrophy, progressive supranuclear palsy, diffusion weighted Imaging

## Abstract

**Introduction:** White matter degeneration may contribute to clinical symptoms of parkinsonism.

**Objective:** We used fixel-based analysis (FBA) to compare the extent and patterns of white matter degeneration in different parkinsonian syndromes—including idiopathic Parkinson's disease (PD), multiple system atrophy (MSA), and progressive supranuclear palsy (PSP).

**Methods:** This is a retrospective interpretation of prospectively acquired data of patients recruited in previous studies during 2008 and 2019. Diffusion-weighted images were acquired on a 3-Tesla scanner (diffusion weighting b = 1000 s/mm^2^–applied along either 64 or 30 non-collinear directions) from 53 patients with PD (men/women: 29/24; mean age: 65.06 ± 5.51 years), 47 with MSA (men/women: 20/27; mean age: 63.00 ± 7.19 years), and 50 with PSP men/women: 20/30; mean age: 65.96 ± 3.14 years). Non-parametric permutation tests were used to detect intergroup differences in fixel-related indices—including fiber density, fiber cross-section, and their combination.

**Results:** Patterns of white matter degeneration were significantly different between PD and atypical parkinsonisms (MSA and PSP). Compared with patients with PD, those with MSA and PSP showed a more extensive white matter involvement—noticeably descending tracts from primary motor cortex to corona radiata and cerebral peduncle. Lesions of corpus callosum were specific to PSP and absent in both MSA and PD.

**Discussion:** FBA identified specific patterns of white matter changes in MSA and PSP patients compared to PD. Our results proved the utility of FBA in evaluation of implied biological processes of white matter changes in parkinsonism. Our study set the stage for future applications of this technique in patients with parkinsonian syndromes.

## Introduction

Parkinsonism is a progressive neurodegenerative disorder characterized by resting tremor, rigidity, bradykinesia/akinesia, and postural instability (McFarland, 2016). Although idiopathic Parkinson's disease (PD) is the most common form of parkinsonism, this condition also comprises multiple system atrophy (MSA) and progressive supranuclear palsy (PSP)—two atypical parkinsonian syndromes that tend to have a more rapid functional deterioration compared with idiopathic PD (McFarland and Hess, 2017). Apart from the pathogenetic changes in basal ganglia, neuroimaging studies in PD have shown various extents of atrophy affecting different brain regions—including a reduced gray matter volume in the frontal (Burton et al., 2004), and pre-frontal lobes (Biundo et al., 2011). Atrophy in the cortical and subcortical areas and noticeably in the cerebellum has been reported in patients with PSP (Giordano et al., 2013), whereas cerebellar white matter atrophy has been described in MSA(Matsusue et al., 2009). Extensive involvement of different brain regions is accompanied by white matter degeneration, which may result in a clinically relevant functional decline (Whitwell et al., 2011).

Although diffusion tensor imaging (DTI) has been previously applied to investigate white matter changes in patients with parkinsonism, this technique suffers from several shortcomings. Erroneous interpretations in DTI may result from oversimplification of the underlying anatomical structures (Mori and Zhang, 2006; Chen et al., 2019), especially in regions with crossing fibers (Jbabdi et al., 2010). In this scenario, fixel-based analysis (FBA) has emerged as a novel approach based on a higher-order diffusion model to compute fiber orientation density function (Raffelt et al., 2017). FBA allows investigating the micro- and macrostructural properties of individual fiber populations within each voxel—with a single fiber population within a voxel termed fixel (Pecheva et al., 2019). Three fixel-based indices can be derived, which are fiber density (FD)—the volume of intra-axonal space of particular fixel; fiber cross-section (FC)—the cross-sectional area of particular fixel; and the combination of fiber density and cross-section (FDC) (Pecheva et al., 2019).

Previous studies supported the clinical usefulness of FBA in investigating white matter degeneration in patients with neurodegenerative diseases (Mito et al., 2018; Rau et al., 2019). Understandings of white matter changes in parkinsonism may provide new insights into the pathogenesis of motor and non-motor symptoms in these clinical entities, which may eventually lead to more personalized care in terms of diagnosis and treatment. The study is original by using FBA, which is a novel development and could provide new interpretation of the underlying changes in the brain as measured by diffusion. We therefore designed this retrospective analysis of prospectively collected data. We used fixel-based analysis to compare the extent and patterns of white matter degeneration in different parkinsonian syndromes—including idiopathic Parkinson's disease, multiple system atrophy, and progressive supranuclear palsy.

## Materials and Methods

This is a retrospective interpretation of prospectively acquired data of diffusion MRI. Both diffusion tensor imaging and structural images (as acquired from T1 weighted MPRAGE sequence) were extracted during the period of 2008 and 2019 from medical records. The study protocol complied with the tenets of the Helsinki declaration, and ethical approval was granted by the Chang Gung Medical Foundation Institutional Review Board. Owing to the retrospective nature of the study, the need for informed consent was waived.

### Patients

Images were obtained from 53 patients with PD (29 men and 24 women; mean age: 65.06 ± 5.51 years), 47 with MSA (20 men and 27 women; mean age: 63.00 ± 7.19 years), and 50 with PSP (20 men and 30 women, mean age: 65.96 ± 3.14 years). Eighty-five participants were involved in prior publications, which focused on the analysis of diffusion tensor imaging and its application in the diagnosis and prognosis of patients with Parkinsonism, as well as longitudinal FBA analysis in patients with PD (Wang et al., 2010, 2011; Wai et al., 2012; Lu et al., 2016; Rau et al., 2019b; Chen et al., 2020; Tsai et al., 2020). The current submission employed patients with PD, MSA, and PSP, which investigated white matter difference by FBA analysis. A list of references was added in the Supplementary Table 1. Patients were clinically diagnosed made by two senior neurologists (CSL and YHW, 28 and 21 years of experience, respectively) according to commonly accepted criteria for PD (Hughes et al., 1992), MSA (Gilman et al., 1999), and PSP (Litvan et al., 1996), respectively. The acquisition should be consisted of both diffusion tensor imaging and high-resolution T1-weighted anatomical images. 99mTc-TRODAT-1, which binds to the dopamine transporter, was used to image the dopaminergic system in all participants. Exclusion criteria were as follows: (1) moderate-to-severe dementia; (2) severe dyskinesia; (3) major systemic medical conditions; (4) documented brain abnormalities based on MRI or ^18^FDG PET findings; (5) history of intracranial surgery; (6) significant neuropsychiatric disorders established by the corresponding diagnostic criteria, including stroke, brain tumor, demyelinating diseases of central nervous system (CNS), major depression, schizophrenia, and Alzheimer's disease; and (7) pharmacotherapy lasting for >10 years or treatment with drugs capable of crossing the blood-brain-barrier (the only exception being medications for parkinsonian syndromes). The Modified Hoehn and Yahr Staging (Goetz et al., 2004) and the motor subscale of Unified Parkinson Disease Rating Scale (UPDRS III) (Martinez-Martin et al., 1994) were used to assess clinical severity.

Two neuro-radiologists (YLC and YMW, 21 and 11 years of experience, respectively), who were blinded to the diagnosis, read the structural MR images independently.

### Data Acquisition

All images were acquired on a 3.0-Tesla scanner (Trio Magnetom; Siemens, Erlangen, Germany) using a 12-channel head coil. Diffusion-weighted imaging was performed using two protocols characterized by a different diffusion-sensitive spin-echo EPI sequence. In brief, images with a diffusion weighting b = 1000 s/mm^2^ were acquired along 64 or 30 non-collinear directions. The voxel size was either 2 × 2 × 2 mm^3^ or 2 × 2 × 3 mm^3^. High-resolution T1-weighted anatomical images were obtained using magnetization-prepared rapid gradient-echo (MPRAGE) sequences with the following parameters: TR, 2000 ms; inversion time (TI), 900 ms; TE, 2.63 ms; voxel size, 1 × 1 × 1 mm^3^.

### Image Processing

FBA was carried out on diffusion MRI using MRtrix 3.0 following the recommendations of Raffelt et al. (2017). Pre-processing included denoising by principal component analysis (Veraart et al., 2016), removal of Gibbs ringing (Kellner et al., 2016), as well as correction for motion, distortion, and bias field (Tustison et al., 2010; Andersson and Sotiropoulos, 2015, 2016). Multi-tissue constrained spherical deconvolution was used to estimate fiber orientations distribution in each voxel (Jeurissen et al., 2014). A study-specific template was created by spatial normalization in all of the study patients using symmetric non-linear transformation fiber orientations distribution-based registration. Fiber density and fiber bundle cross-section were calculated within each voxel. A combined measure of fiber density and cross-section (FDC) was computed by multiplying FD by FC (10). For comparison, both Fractional Anisotropy (FA) and Mean Diffusivity (MD) from diffusion tensor imaging were analyzed by using Tract Based Spatial Statistics (TBSS) (Smith et al., 2006) following the recommended procedure.

### Statistical Analysis

Differences in fixel-related indices between the three study groups were assessed using non-parametric permutation testing and connectivity-based fixel enhancement as implemented in MRtrix 3.0. Age, sex, and different imaging protocol was used as potential confounding factors. A family-wise error corrected *p-*value < 0.05 was considered statistically significant (Nichols and Holmes, 2002).

## Results

### Demographic and Clinical Variables

Table 1 depicts the general characteristics of the study patients. Age and sex did not differ significantly among the three study groups. Disease duration was longer in patients with PD than in those with MSA (*p* = 0.001). UPDRS III scores were significantly higher in the two atypical parkinsonian syndromes than in patients with PD (*p* < 0.001).

**Table 1:** Demographic and clinical variables.

	**PD**	**MSA**	**PSP**	***p*-value**
Number of subject	53	47	50	
Sex (M/F)	29/24	20/27	20/30	0.277
Age (years)	65.1 ± 5.5	63.0 ± 7.2	66.0 ± 3.1	0.069
Duration (months)	77.9 ± 52.5^a^	49.3 ± 31.4	62.9 ± 37.7	0.004
MHY				<0.001
1	15	0	0	
1.5	4	0	1	
2	8	1	1	
2.5	10	2	2	
≥3	12	29	46	
UPDRS III	22.8 ± 15.2^b,c^	37.2 ± 17.2	37.3 ± 17.8	<0.001
N/A*	4	15	0	

* *Data was not available in both MHY and UPDRS III. PD, Idiopathic Parkinson's disease; MSA, Multiple system atrophy; PSP, Progressive supranuclear palsy; MHY, Modified Hoehns and Yahr staging scale; UPDRS III, Motor subscale of Unified Parkinson's Disease Rating Scale*.

a *PD vs. MSA, p = 0.001*.

b *PD vs. MSA, p < 0.001*.

c *PD vs. PSP, p < 0.001*.

### Fixel-Related Indices in the Three Study Groups

**Figure 1:**
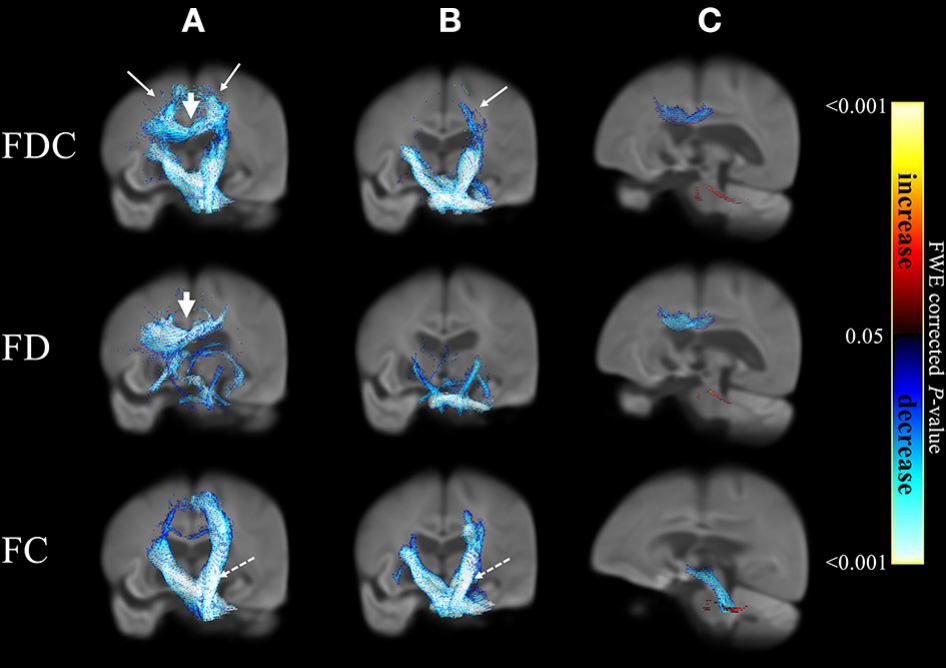
Differences in fixel-related indices in PSP compared to PD (column a), MSA compared to PD (column b) and PSP compared to MSA (column c). The involved regions included corona radiata (white arrows in column a and column b) and cerebral peduncle (dashed arrows in columns a, b). Corpus callosum is specifically involved when compared PSP to PD (arrowhead in column a). In PSP group when compared to MSA (column c), the regions with reduced FDC and FD were mainly located in corpus callosum and regions with reduced FC can be found in midbrain and cerebral peduncles (blue). In MSA group when compared to PSP (column c), the regions with reduced FDC, FD and FC were located in middle cerebellar peduncles (red). The streamline showed *p*-values (family-wise error corrected *p* < 0.05, indicated by the colorbar).

Figure 1 displays the changes in fixel-related indices in patients with atypical parkinsonian syndromes (column a: PSP; column b: MSA) compared with PD. All indices were consistently reduced. A 3D visualization of the affected regions with the color encoded the direction of the major fiber bundles are shown in Supplementary Figure 1 (PSP) and 2 (MSA), respectively. With respect to PD, atypical parkinsonian syndromes were characterized by a significant involvement of white matter tract from corona radiata (white arrows in columns a and b) to cerebral peduncle (dashed arrows in columns a and b). Body of corpus callosum was found to be affected when PSP was compared with PD (arrow head in column a).

A comparison of PSP and MSA revealed a differential involvement of various brain areas (column c). Supplementary Figure 3 showed the 3D visualization of the affected regions (color blue: reduction in PSP; color red: reduction in MSA). Regions characterized by reduced FDC (upper row) and FD (middle row) in patients with PSP were chiefly located in the body of corpus callosum, whereas midbrain and cerebral peduncles showed a reduction in FC (blue, bottom row). Regions displaying reduced FDC, FD, and FC values in patients with MSA were localized in the superior cerebellar peduncles (red).

### Comparison of Patients With PSP and PD

**Figure 2:**
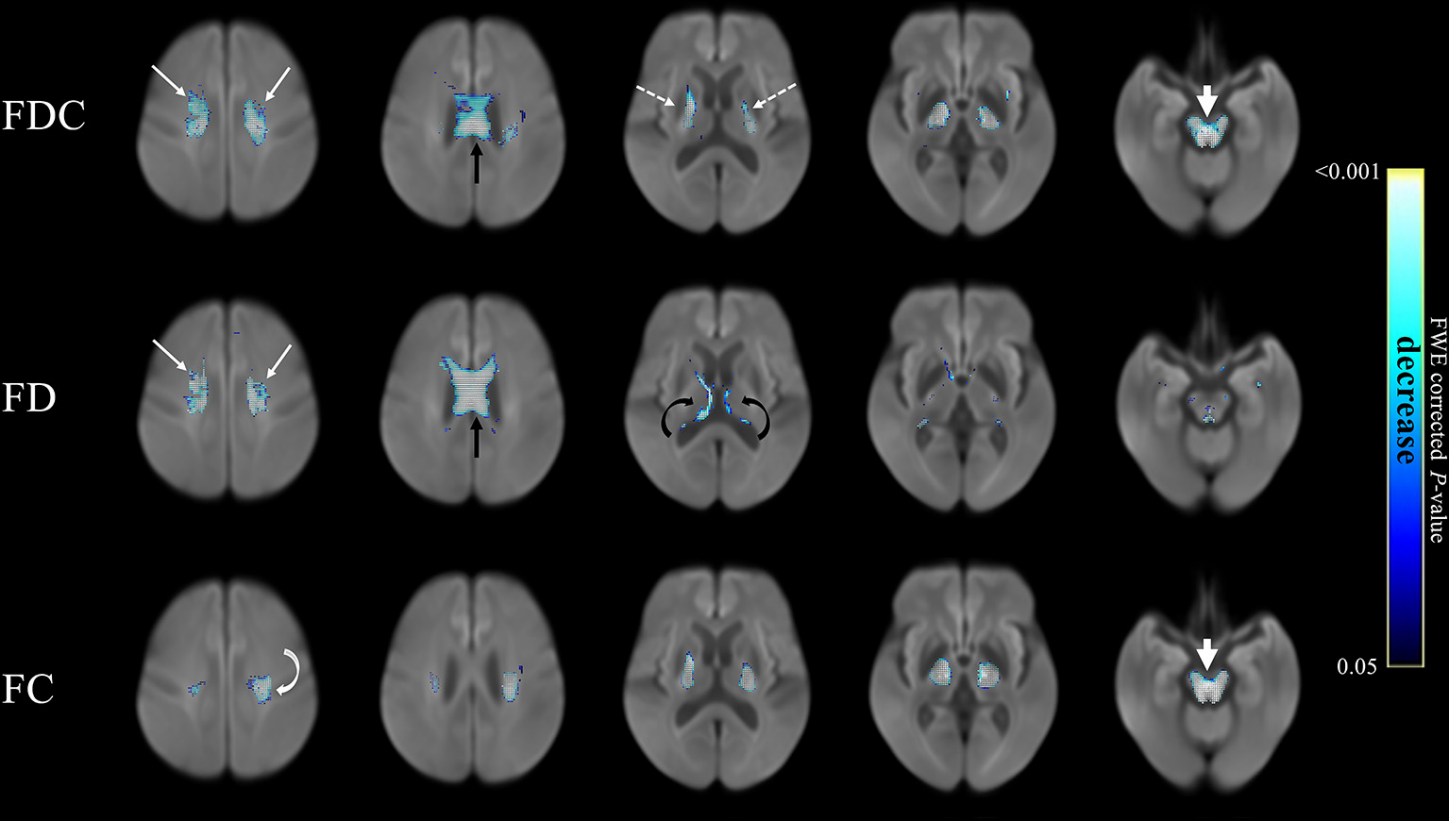
Fixel-related indices in patients with PSP compared to that with PD. Reduction of FDC in the main white matter tracts, from superior region of corona radiata (upper row, white arrows), corpus callosum (upper row, black arrow) to posterior limbs of internal capsules (upper row, dashed arrows) and cerebral peduncles (upper row, arrowhead). Reduced FD was identified in corona radiata (middle row, white arrows), corpus callosum (middle row, black arrow) and the fornix (middle row, black curved arrows), which were symmetrical in the supratentorial compartment. Reduced FC was noticed in corona radiata to posterior limbs of internal capsules (bottom row, white curved arrow) and cerebral peduncles (bottom row, white arrowhead). The streamline showed *p*-values (family-wise error corrected *p* < 0.05, indicated by the colorbar).

Figure 2 compares fixel-related indices of patients with PSP and PD. Reductions of FDC (upper row) were evident in the main descending white matter tracts from the superior region of corona radiata (from motor cortex) (white arrows) through body of corpus callosum (black arrow) to posterior limbs of internal capsules (dashed arrows) and into the cerebral peduncles (arrow head). The regions with changes are consistent with that of reduced FD (middle row), noticeably in corona radiata (white arrows), and corpus callosum (black arrow). There was involvement of the medial thalamus (black curved arrow)—with a symmetrical involvement of the supratentorial compartment. Similar regions of reductions in FC (bottom row) were evident from corona radiata to the posterior limbs of internal capsules and cerebral peduncles (arrow head). Regions with a concomitant reduction of both FD and FC were generally overlapping to those with a reduced FDC. However, an isolated decreased of FD was evident in the thalamus. The names of tracts with significant difference, together with the cluster sizes and the peak *p*-values were summarized in Table 2.

**Table 2:** Comparison of fiber tracts between patients with PSP and PD.

**Fixel-related indices**	**Name of tracts**	**Cluster size**	**Peak *p*-value**
FDC	Body of corpus callosum	2532	0.00020
	Posterior limb of internal capsule L	1025	0.00020
	Superior corona radiata L	992	0.00040
	Posterior limb of internal capsule R	896	0.00020
	Superior corona radiata R	743	0.00040
	Cerebral peduncle R	658	0.00020
	Cerebral peduncle L	643	0.00020
	Anterior limb of internal capsule R	259	0.00040
	Superior cerebellar peduncle L	251	0.00020
	Superior cerebellar peduncle R	246	0.00020
	Corticospinal tract L	204	0.00180
	Pontine crossing tract	190	0.00020
	Corticospinal tract R	137	0.00180
	Medial lemniscus L	86	0.00020
	Medial lemniscus R	63	0.00040
	Anterior limb of internal capsule L	54	0.01440
	External capsule R	39	0.02460
	Crura of fornix R	31	0.02620
FD	Body of corpus callosum	3130	0.00020
	Superior corona radiata R	489	0.00080
	Superior corona radiata L	271	0.00100
	Crura of fornix R	171	0.00160
	Superior cerebellar peduncle R	162	0.00200
	Crura of fornix L	152	0.00720
	Superior cerebellar peduncle L	88	0.00300
	Cerebral peduncle L	82	0.01700
	Cerebral peduncle R	78	0.02600
	Anterior limb of internal capsule R	63	0.00500
	Posterior limb of internal capsule L	55	0.02100
	Genu of corpus callosum	33	0.02920
	Posterior limb of internal capsule R	27	0.02400
	Medial lemniscus R	21	0.01200
	Corticospinal tract R	14	0.03300
	Corticospinal tract L	14	0.02100
	Medial lemniscus L	11	0.02800
FC	Superior corona radiata L	1549	0.00020
	Posterior limb of internal capsule L	1194	0.00020
	Posterior limb of internal capsule R	1086	0.00820
	Cerebral peduncle L	611	0.00020
	Cerebral peduncle R	564	0.00020
	Superior corona radiata R	552	0.00020
	Superior cerebellar peduncle L	261	0.00020
	Body of corpus callosum	258	0.00040
	Pontine crossing tract	235	0.00080
	Corticospinal tract L	234	0.00500
	superior cerebellar peduncle R	231	0.00020
	posterior corona radiata L	131	0.00340
	anterior limb of internal capsule R	127	0.00020
	corticospinal tract R	109	0.00500
	middle cerebellar peduncle	103	0.00220
	medial lemniscus L	82	0.00020
	medial lemniscus R	58	0.00060
	inferior cerebellar peduncle L	23	0.00200
	anterior limb of internal capsule L	11	0.00140
	inferior cerebellar peduncle R	10	0.00020

### Comparison of Patients With MSA and PD

**Figure 3:**
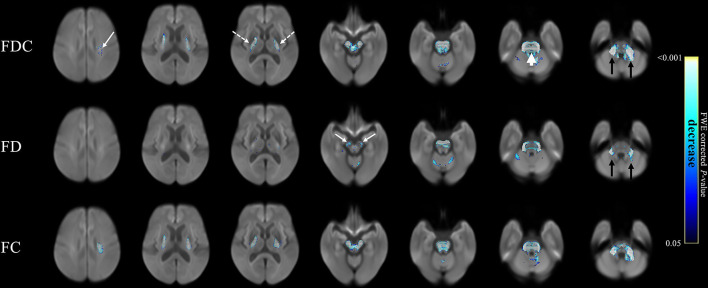
Fixel-related indices in patients with MSA compared to that with PD. Reduced FDC were identified in the main descending white matter pathways, from left corona radiata (upper row, white arrow) to bilateral posterior limbs of internal capsule (upper row, white dashed arrows), cerebral peduncles, transvers pontine fibers (upper row, white arrowhead), and bilateral middle cerebellar peduncles (upper row, black arrows). Reduce FD was found in bilateral cerebral peduncles (middle row, white arrows) and middle cerebellar peduncles (middle row, black arrows). Left corona radiate and middle cerebellar peduncle are more affected than the right side. The streamline showed *p*-values (family-wise error corrected *p* < 0.05, indicated by the colorbar).

Figure 3 compares fixel-related indices of patients with MSA and PD. Reductions of FDC (top row) were evident in the main descending white matter pathways from the left corona radiata (white arrows) to bilateral posterior limbs of internal capsule (white dashed arrows), cerebral peduncles, transverse pontine fibers (white arrow head), and bilateral middle cerebellar peduncles (black arrows). FD was found to be significantly reduced (middle row) in bilateral cerebral peduncles (white arrows) and middle cerebellar peduncles (black arrows). Interestingly, regions with reduced FC (bottom row) almost invariably overlap with those showing a reduction in FDC. The names of tracts with significant difference, together with the cluster sizes and the peak *p*-values were summarized in Table 3.

**Table 3:** Comparison of fiber tracts between patients with MSA and PD.

**Fixel-related indices**	**Name of tracts**	**Cluster size**	**Peak *p*-value**
FDC	Posterior limb of internal capsule L	1027	0.00020
	Posterior limb of internal capsule R	931	0.00020
	Middle cerebellar peduncle	800	0.00020
	Cerebral peduncle R	768	0.00020
	Cerebral peduncle L	701	0.00020
	Superior corona radiata L	378	0.00760
	Corticospinal tract L	321	0.00020
	Corticospinal tract R	305	0.00020
	Pontine crossing tract	293	0.00020
	Superior cerebellar peduncle R	178	0.00180
	Superior cerebellar peduncle L	149	0.00680
	Anterior limb of internal capsule R	125	0.00060
	Medial lemniscus L	83	0.00320
	Medial lemniscus R	58	0.00200
	Retrolenticular part of internal capsule L	52	0.00080
	Posterior corona radiata L	52	0.02280
	Inferior cerebellar peduncle L	49	0.00120
	Retrolenticular part of internal capsule R	49	0.00180
	Anterior limb of internal capsule L	29	0.01380
	Superior corona radiata R	14	0.01500
FD	Middle cerebellar peduncle	411	0.00020
	Cerebral peduncle R	368	0.00020
	Cerebral peduncle L	273	0.00040
	Posterior limb of internal capsule R	192	0.00660
	Corticospinal tract L	171	0.00040
	Posterior limb of internal capsule L	135	0.00620
	Corticospinal tract R	132	0.00140
	Pontine crossing tract	67	0.00640
	Retrolenticular part of internal capsule R	29	0.00600
	Superior_part_cingulum_R	27	0.02400
	Superior cerebellar peduncle R	24	0.02000
	Crura of fornix L	17	0.03800
	Crura of fornix R	14	0.02900
FC	Posterior limb of internal capsule L	1139	0.00020
	Posterior limb of internal capsule R	1024	0.00020
	Superior corona radiata L	991	0.00100
	Middle cerebellar peduncle	792	0.00020
	Cerebral peduncle R	662	0.00020
	Cerebral peduncle L	633	0.00020
	Corticospinal tract L	334	0.00020
	Corticospinal tract R	326	0.00020
	Pontine crossing tract	269	0.00020
	Superior cerebellar peduncle L	124	0.00460
	Posterior corona radiata L	117	0.00700
	Superior corona radiata R	96	0.01180
	Anterior limb of internal capsule R	71	0.00340
	Superior cerebellar peduncle R	66	0.00480
	Inferior cerebellar peduncle L	56	0.00440
	Medial lemniscus L	21	0.00700
	Anterior limb of internal capsule L	18	0.00700

### Comparison of Patients With PSP and MSA

**Figure 4:**
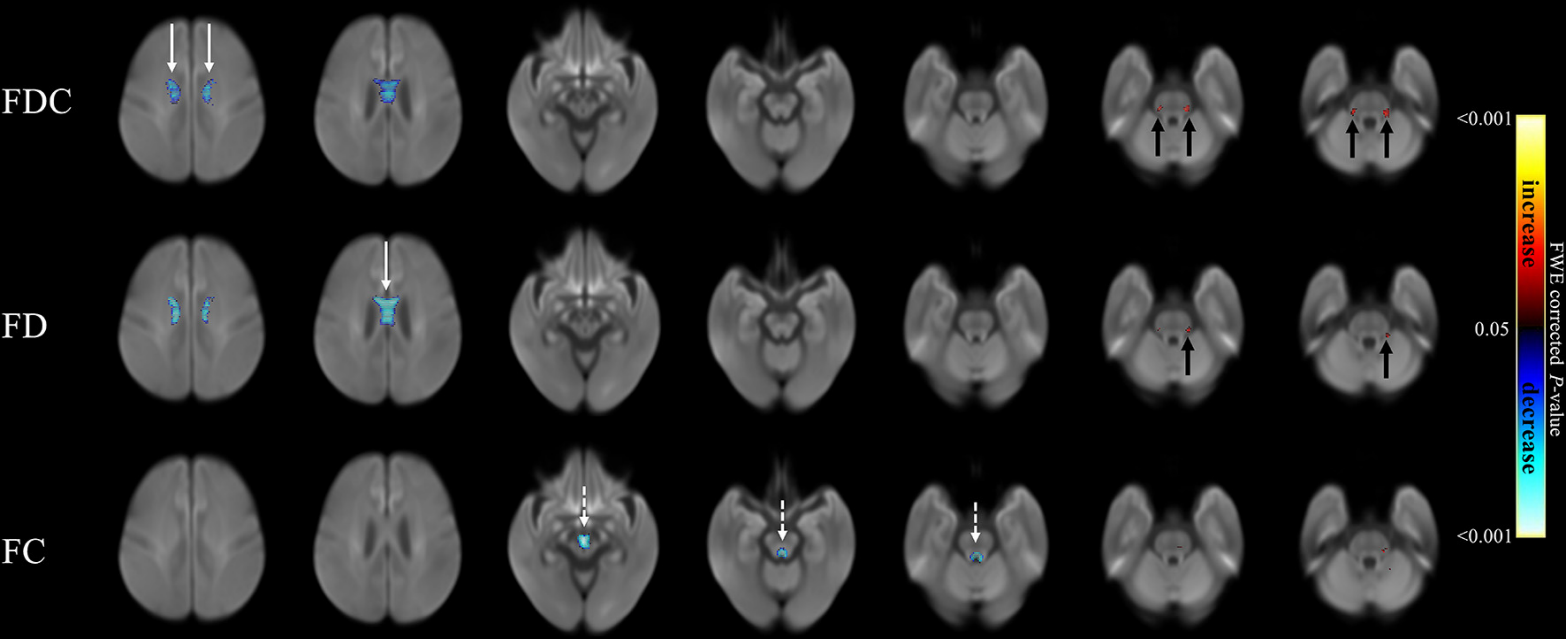
Fixel-related indices in patients with PSP and MSA. Reduced FDC and FD were noticeable in corpus callosum (white arrows), while reduced FC were found in midbrain (white dashed arrows) in patients with PSP. On the other hand, the regions with reduced FDC, FD and FC were found in middle cerebellar peduncles (black arrows) in patients with MSA. The streamline showed *p*-values (family-wise error corrected *p* < 0.05, indicated by the colorbar).

Figure 4 compares fixel-related indices of patients with PSP and MSA. Reductions of FDC and FD in patients with PSP were evident in body of corpus callosum (white arrows), whereas FC was found to be lowered in the midbrain (white dashed arrows). All fixel-related indices (FDC, FD, and FC) of patients with MSA were reduced in middle cerebellar peduncles (Figure 4, black arrows). The names of tracts with significant difference, together with the cluster sizes and the peak *p*-values were summarized in Table 4.

**Table 4:** Comparison of fiber tracts between patients with PSP and MSA.

**Fixel-related indices**	**Name of tract**	**Cluster size**	**Peak *p*-value**
FDC	Body of corpus callosum	1048	0.01360
	Middle cerebellar peduncle	118	0.03000
	Superior corona radiata R	110	0.01340
	Corticospinal tract L	40	0.02140
	Superior corona radiata L	35	0.01700
	Corticospinal tract R	25	0.02740
	Cerebral peduncle L	11	0.02400
FD	Body of corpus callosum	1196	0.00640
	Superior corona radiata R	55	0.01040
	Superior corona radiata L	22	0.02940
	Corticospinal tract L	18	0.02680
	Cerebral peduncle L	17	0.02000
	Middle cerebellar peduncle	13	0.03260
	Corticospinal tract R	10	0.03080
FC	Middle cerebellar peduncle	58	0.03160
	Superior cerebellar peduncle R	48	0.01680
	Superior cerebellar peduncle L	45	0.01340
	Medial lemniscus L	38	0.01080
	Medial lemniscus R	32	0.01680

### Tract-Based Spatial Statistics in Patients With PSP or MSA, as Compared to PD

**Figure 5:**
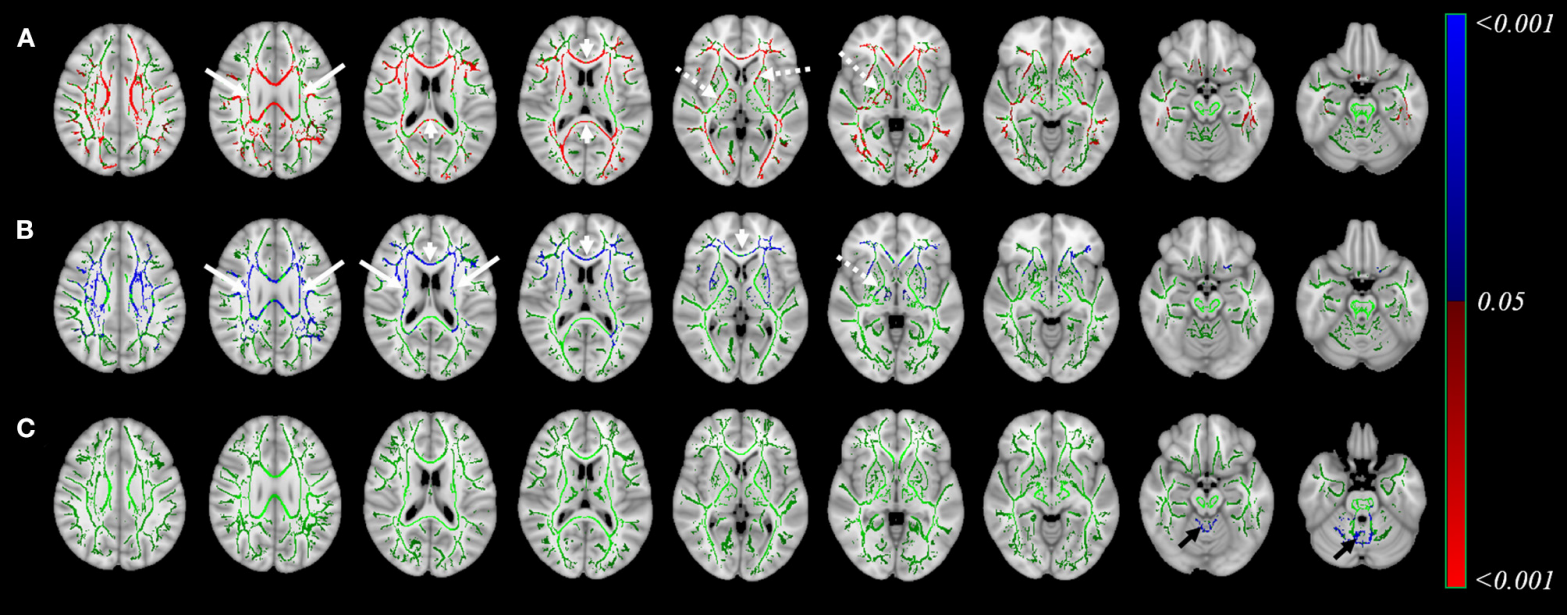
Result of Tract-Based Spatial Statistics in patients with PSP or MSA as compared to PD. This figure shows the analysis from tract-based spatial statistics in patients with PSP or MSA, when compared to PD. Reduced fractional anisotropy (FA, Result of Tract-Based Spatial Statistics in patients with PSP or MSA as compared to PD. This figure shows the analysis from tract-based spatial statistics in patients with PSP or MSA, when compared to PD. Reduced fractional anisotropy (FA, A) and increased mean diffusivity (MD, B) in patients with PSP was found in bilateral corona radiate (arrows), internal capsules (dashed arrow), superior longitudinal fasciculi, posterior thalamic radiation, as well as genu and splenium of the corpus callosum (arrowhead). Increased MD in patients with MSA (C) was found in in bilateral cerebellar peduncle (black arrows). Color indicated the *p*-value, with color code included. Color Green: the identified white matter skeleton.) and increased mean diffusivity (MD, **B**) in patients with PSP was found in bilateral corona radiate (arrows), internal capsules (dashed arrow), superior longitudinal fasciculi, posterior thalamic radiation, as well as genu and splenium of the corpus callosum (arrowhead). Increased MD in patients with MSA **(C)** was found in in bilateral cerebellar peduncle (black arrows). Color indicated the *p*-value, with color code included. Color Green: the identified white matter skeleton.

For comparison to conventional diffusion tensor imaging, Figure 5 shows the result from tract-based spatial statistics relative to patients with PD. In patients with PSP, reduced FA (panel a) and increased MD (panel b) can be found in bilateral corona radiata, internal capsules, superior longitudinal fasciculi, posterior thalamic radiation, as well as genu and splenium of the corpus callosum. In patients with MSA, only increased MD (panel c) was found, which was located in the bilateral cerebellar peduncle.

## Discussion

### Main Findings

FBA was used to assess the patterns of white matter changes in three different forms of parkinsonism. Our findings revealed profound differences in terms of white matter involvement among groups—with affected regions being more extensive in MSA and PSP than in PD. These results are consistent with the notion that both PSP and MSA portend a higher burden of clinical disability. Further, brain regions affected by white matter changes were in accordance with the current models of disease pathogenesis (Boxer et al., 2017; Meissner et al., 2019). Our analysis method might provide an interesting insight into the pathophysiology of various parkinsonisms. Taken together, our data indicate that the distribution and severity of white matter involvement in patients with PSP and MSA are characteristically different.

The identified regions can be overlapped between FBA and conventional DTI analysis using TBSS. Both techniques might provide complementary information regarding to the underlying microstructural changes. However, because different post-processing procedures may lead to either false-positive or false-negative results (Kuchling et al., 2018), the choice of the analysis approach might depend on the research question of interest. Our study demonstrated the use of fixel-related indices as potential biomarkers of disease progression. It may set the stage for future applications of this technique in patients with parkinsonian syndromes.

### PSP: From Corpus Callosum, Internal Capsules, Thalamic Radiation to Midbrain

The corpus callosum consists of white matter fibers that interconnect the motor areas of the two hemispheres. When compared to patients with PD, reductions of FDC in the corpus callosum of PSP overlapped considerably with FD declines. Similarly, when compared to patients with MSA, a reduced FDC in the corpus callosum can be noticed, a change which was chiefly driven by a decline in FD rather than in FC. Reductions of FD may be related to a lowered intra-axonal volume as a result of axonal degeneration (Raffelt et al., 2017). In this scenario, our findings might reflect the occurrence of Wallerian degeneration in the affected descending pathways–possibly secondary to cortical gray matter atrophy (Worker et al., 2014). Our findings support the early-stage white matter involvement in corpus callosum in PSP, which may aid in our understanding of the difference in pathogenesis between PSP and MSA.

Previous studies have reported that patients with PSP display abnormal diffusion metrics in the main white matter tract in addition to corpus callosum—for example, the internal capsule (Padovani et al., 2006; Agosta et al., 2012). Our findings of altered fixel-related indices in these regions suggest that the previously reported abnormalities are likely the results of microstructural white matter injury. Patients with PSP have a reduced FDC in both the corona radiata and internal capsules. Such alterations are in line with the higher severity of motor deficits observed in patients with PSP than in those with PD (Alster et al., 2020).

Notably, regions with reduced FDC in the corona radiata, internal capsules, and midbrain had broad overlaps with those showing diminished FC. The latter alteration can be attributed to a narrowing of extra-axonal space followed by axonal loss and disruption of myelin sheaths as a result of white matter atrophy (Raffelt et al., 2017). Interestingly, regions with reduced FD was found in bilateral medial thalami. These fibers are parts of thalamic radiation which connect the thalami with the cortex via internal capsule (Sun et al., 2018). Reduced FD, rather than FC, might imply early changes within these fibers. Similar regions have been reported in a quantitative study of histology, which showed vacuolation and glial inclusion in descending white matter tract from frontal cortex through the internal capsules and midbrain (Armstrong, 2013). Our findings suggest the involvement of the thalamic radiation in PSP, which may help distinguishing this disease from PD and MSA.

Taken together, these results suggest that the severity of white matter damage in internal capsules, thalamic radiation and midbrain in PSP is more pronounced than in PD. Our findings suggest that the previously reported abnormalities are likely the results of microstructural white matter injury.

### MSA: Corona Radiata, Internal Capsule, and Middle Cerebellar Peduncles

MSA is specifically characterized by an involvement of the middle cerebellar peduncles (Poewe and Wenning, 2002). When patients with MSA were compared to those with PD, we found significant reductions of both FDC and FC in the main descending white matter pathways. A previous DTI study identified large areas characterized by reduced FA and increased MD in white matter tracts—especially in the supratentorial and infratentorial compartments (Tha et al., 2010). Here, we provide evidence of a substantial axonal loss in the white matter located in these areas.

When compared with those with PD as well as PSP, we found significant reductions of FDC in the middle cerebellar peduncles—which was accompanied by parallel declines in both FD and FC. Increased diffusivity has been previously reported in this region (Tsukamoto et al., 2012). Our results indicate that these regions, characterized by a reduced FDC, were driven by a concomitant decline in both FD and FC suggesting that axonal degeneration is likely the main contributor to microstructural damage. MSA can be associated with a more severe axonal neurodegeneration and white matter atrophy in these areas compared with either PD or PSP. Previous postmortem pathology study showed that the white matter atrophy of the cerebellum in patients with MSA can be attributed to the loss of myelinated fibers and gliosis (Matsusue et al., 2009b). Our observation of reduced FD and FC, as explained by the simultaneous presence of axonal loss and white matter atrophy, might ultimately reflect more severe derangements as related to the motor deficits in patients with MSA than in those with PD.

Significant reductions of both FDC and FC were also found in the main descending white matter pathways, namely the left corona radiata, bilateral posterior limbs of internal capsule, cerebral peduncles, and ventral and dorsal transverse pontine fibers. Concomitant reduction of all three fixel-related indices in these regions implies severe white matter damages. This is in line with the more severe morbidity of MSA in comparison to PSP and PD. Here, we provide image-based evidence of a substantial axonal loss in the white matter located in these areas.

### Limitation

Our findings should be interpreted in the context of some limitations. In this study, patients with PD was used as a “parkinsonian control” group with which both the patients with MSA or PSP were compared to. In the future study when using a prospective design, we will recruit the healthy control in our study for a comprehensive understanding of the white matter involvement in Parkinsonism. Secondly, it may be argued that the study groups were different in terms of imaging protocols. However, the effect from this variable, together with age and sex, has been considered and controlled in the statistical analysis. Provided the acquisition and the analysis pipeline are the same, these measures can be quantifiable to allow for assessment of disease severity within study. However, it would be necessary to perform advanced statistical analysis to determine the sensitivity and specificity before its potential application in early-stage therapeutic trials, or to monitor disease progression. More radiologic—pathologic correlation studies are necessary to scrutinize the biologic mechanisms of these fixel-related indices' alteration. These caveats notwithstanding, we successfully applied FBA and identified specific patterns of white matter degeneration that distinguished PD from atypical parkinsonisms (MSA and PSP).

## Data Availability Statement

The data that support the findings of this study are available from the corresponding author upon reasonable request.

## Ethics Statement

The studies involving human participants were reviewed and approved by the study protocol complied with the tenets of the Helsinki declaration, and ethical approval was granted by the Chang Gung Medical Foundation Institutional Review Board. Owing to the retrospective nature of the study, the need for informed consent was waived. Written informed consent for participation was not required for this study in accordance with the national legislation and the institutional requirements.

## Author Contributions

T-TN: conception and design of study, acquisition of clinical data, and writing and revision of the manuscript. J-SC: conception and design of study, statistical analysis, and writing the first draft. Y-LC, Y-CL, C-SL, Y-HW, and Y-MW: acquisition of clinical data and revision the manuscript. C-CT: statistical analysis and revision the manuscript. N-TH: revision the manuscript. J-JW: conception and design of study, writing and revision the manuscript, study supervision, and obtaining funding. All authors contributed to the article and approved the submitted version.

## Conflict of Interest

The authors declare that the research was conducted in the absence of any commercial or financial relationships that could be construed as a potential conflict of interest.
