# Supplementary material for: Fixel-Based Analysis of White Matter Degeneration in Patients With Progressive Supranuclear Palsy or Multiple System Atrophy, as Compared to Parkinson's Disease
Source: Front Aging Neurosci. 2021 Mar 16;13:625874. doi: 10.3389/fnagi.2021.625874 (PMC8018443; doi:10.3389/fnagi.2021.625874)
Supplement: Supplementary file 1 [file Table_1.docx]

| **Supplementary Table 1. Information and references for the participants in previous studies** | | | | | |
| --- | --- | --- | --- | --- | --- |
| **First author** | **Year** | **Title** | **Total participants** | **Participants in this study** | **DOI** |
| Tsai CC | 2020 | A Method for the Prediction of Clinical Outcome Using Diffusion Magnetic Resonance Imaging: Application on Parkinson's Disease | PD: 82 | PD:16 | 10.3390/jcm9030647 |
| Rau YA | 2019 | A longitudinal fixel-based analysis of white matter alterations in patients with Parkinson's disease | NL: 76 PD: 50 | PD: 10 | 10.1016/j.nicl.2019.102098 |
| Chen YL | 2019 | Prediction of the clinical severity of progressive supranuclear palsy by diffusion tensor imaging | PSP: 53 | PSP: 50 | 10.3390/jcm9010040 |
| Lu CS | 2016 | Alterations of diffusion tensor MRI parameters in the brains of patients with Parkinson's disease compared with normal brains: possible diagnostic use | NL: 91 PD: 126 | PD: 18 | 10.1007/s00330-016-4232-7 |
| Wai YY | 2012 | Cortical involvement in a gait-related imagery task: comparison between Parkinson's disease and normal aging | NL: 27 PD: 13 | PD: 10 | 10.1016/j.parkreldis.2012.02.004 |
| Wang JJ | 2011 | Parkinson disease: diagnostic utility of diffusion kurtosis imaging | NL: 30 PD: 30 | PD: 19 | 10.1148/radiol.11102277 |
| Wang JJ | 2010 | Microstructural changes in patients with progressive supranuclear palsy: A diffusion tensor imaging study | NL: 17 PSP: 17 | PSP: 17 | 10.1002/jmri.22229 |

**Appendix**
